# Advanced Assessment of Oxidative Stress and Inflammation in Military Personnel: Development of a Novel IIRPM Score Using Artificial Intelligence

**DOI:** 10.3390/diagnostics15070832

**Published:** 2025-03-25

**Authors:** Florina-Diana Mihai, Emil-Tiberius Trasca, Patricia-Mihaela Radulescu, Razvan Mercut, Elena-Irina Caluianu, Eleonora Daniela Ciupeanu-Calugaru, Dan Marian Calafeteanu, Georgiana-Andreea Marinescu, Suzana Danoiu, Dumitru Radulescu

**Affiliations:** 1 UMF Craiova Doctoral School, University of Medicine and Pharmacy Craiova, 200349 Craiova, Romania; staicu_diana2002@yahoo.com (F.-D.M.); marinescu_georgiana.andreea@yahoo.com (G.-A.M.); 2 Department of Surgery, University of Medicine and Pharmacy Craiova, 200349 Craiova, Romania; irina.caluianu@umfcv.ro (E.-I.C.); dr_radulescu_dumitru@yahoo.com (D.R.); 3 The Military Emergency Clinical Hospital, ‘Dr. Stefan Odobleja’ Craiova, 200749 Craiova, Romania; danutcalafeteanu@yahoo.com; 4 Department of Pneumology, University of Medicine and Pharmacy Craiova, 200349 Craiova, Romania; 5 Department of Plastic and Reconstructive Surgery, University of Medicine and Pharmacy of Craiova, 200349 Craiova, Romania; mercut.razvan@umfcv.ro; 6 Department of Biology and Environmental Engineering, University of Craiova, 200585 Craiova, Romania; ciupeanudaniela@gmail.com; 7 Department of Ortopedics, University of Medicine and Pharmacy Craiova, 200349 Craiova, Romania; 8 Department of Pathophysiology, University of Medicine and Pharmacy of Craiova, 200349 Craiova, Romania; suzanadanoiu@yahoo.com

**Keywords:** oxidative stress, systemic inflammation, military personnel, integrative inflammatory risk post-mission index (IIRPM), ΔNLR (Delta NLR), neutrophil-to-lymphocyte ratio (NLR), artificial intelligence (AI), machine learning, risk stratification, military medicine

## Abstract

**Background/Objectives:** The health of military personnel in modern operational settings is critical for sustaining defense readiness. Extended exposure to extreme conditions can cause oxidative stress and systemic inflammation, potentially affecting performance. To address this problem, we developed an innovative diagnostic tool, the Post-Mission Integrated Risk Index (IIRPM), which integrates hematologic markers with key clinical variables. A novel aspect of the approach is the incorporation of ΔNLR, thus quantifying the change in the neutrophil-to-lymphocyte ratio measured before and after deployment, thereby providing a sensitive indicator of the inflammatory impact of operational stress. **Methods:** In this retrospective study, we analyzed comprehensive clinical and biological data from 443 military personnel over a ten-year period, with measurements taken before and after missions. We applied robust statistical techniques, including paired *t*-tests and Pearson correlation analyses, to assess variations in hematologic and metabolic parameters. Data segmentation was performed using Gaussian mixture models, and the predictive performance of the resulting model was validated with a multi-layer perceptron (MLP) neural network. **Results:** The analysis revealed significant post-mission increases in both the baseline NLR and ΔNLR, accompanied by notable shifts in metabolic markers. Data segmentation identified three distinct profiles: a reference profile characterized by stable immunologic parameters, an acute inflammatory response profile, and a proinflammatory metabolic profile marked by elevated cholesterol levels and higher mean age. Remarkably, the MLP model achieved 100% accuracy on the test set, with an average cross-validation accuracy of 97%. **Conclusions:** The IIRPM—which incorporates ΔNLR, age, mission duration, and cholesterol levels—offers a novel strategy to assess inflammatory risk among military personnel, thus facilitating personalized preventive interventions. Further validation in multicenter and longitudinal studies is anticipated to consolidate the clinical utility of this tool, ultimately fostering a more adaptive approach in military medicine to address the complex challenges of modern operational theaters.

## 1. Introduction

In contemporary operational settings, the well-being of military personnel is paramount to maintaining an effective defense capability. Service members are frequently exposed to environments characterized by extreme physical demands, intense stress, and hostile conditions. Prolonged exposure to such adverse factors, including chronic sleep deprivation, excessive physical exertion, and significant psychological stress, can initiate oxidative stress and systemic inflammation. These processes, when sustained over time, have the potential to compromise both operational performance and rapid response capabilities. Consequently, continuous and comprehensive health monitoring is essential to prevent long-term physiological decline and maintain operational readiness [1,2].

Traditional assessment methodologies, which typically emphasize isolated measurement of individual biomarkers, often fail to capture the complex interplay between the immune response and oxidative stress. However, recent investigations have illuminated the value of integrated hematological indices, such as the neutrophil-to-lymphocyte ratio (NLR), platelet-to-lymphocyte ratio (PLR), and monocyte-to-lymphocyte ratio (MLR) as reliable indicators of systemic inflammation and oxidative stress. In particular, the NLR has emerged as a sensitive and specific marker of inflammation, closely associated with an overproduction of reactive oxygen species that can disrupt cell integrity by overwhelming endogenous antioxidant defenses [3,4,5]. Elevated NLR values are not only indicative of acute inflammatory responses but also signal an increased predisposition to chronic diseases, including cardiovascular disease [6,7,8,9,10]. Integrating these hematological markers with critical clinical parameters such as age, duration of mission, and cholesterol levels offers the promise of a comprehensive risk assessment, allowing personalized and preventive interventions.

Despite these advances, conventional biomarker assessments are often hampered by their fragmented and labor-intensive nature, which hinders rapid and holistic data interpretation. The absence of a standardized framework for data integration further complicates the comparability of results across studies and may lead to an underestimation of systemic risk factors. In this context, the incorporation of artificial intelligence (AI) methodologies emerges as a promising solution. Machine learning techniques—exemplified by Gaussian mixture models (GMM) and multi-layer perceptron (MLP) neural networks—are capable of processing large datasets concurrently, uncovering complex patterns, and building predictive models with exceptional precision [11,12]. By synergistically correlating hematological parameters with clinical and demographic information, these AI-driven approaches facilitate a more comprehensive assessment of inflammatory status and provide a robust framework for the ongoing monitoring of military health.

Consequently, the main objective of this study is to develop and validate the IIRPM Score, a composite metric designed to quantify post-mission inflammatory risk. This integrative score combines measures of NLR, PLR, and MLR with clinical variables such as age, duration of mission, and cholesterol levels to allow for precise risk stratification. Using artificial intelligence capabilities, the approach seeks to minimize human error and detect early patterns of inflammatory response, thereby supporting the implementation of customized preventive strategies. The adoption of such an integrated model holds significant promise in improving operational performance and strengthening resilience against extreme stressors [1,13,14,15,16]. Furthermore, the convergence of molecular biology, precision medicine, and AI-driven IT solutions paves the way for the personalization of preventive and therapeutic strategies, informed by the unique profiles of individual military personnel and a refined understanding of adaptive mechanisms to operational stress.

## 2. Materials and Methods

### 2.1. Study Design

This retrospective investigation was carried out at the Military Hospital “Dr. Ștefan Odobleja” in Craiova over a decade-long period, from 1 October 2014 to 31 October 2024. The primary objective was to conduct an in-depth assessment of oxidative stress and inflammation in military personnel. To effectively capture the impact of operational environmental changes, the study focused exclusively on service members deployed on international missions. Clinical and biological data were collected systematically at both the time of deployment and after return.

**Figure 1:**
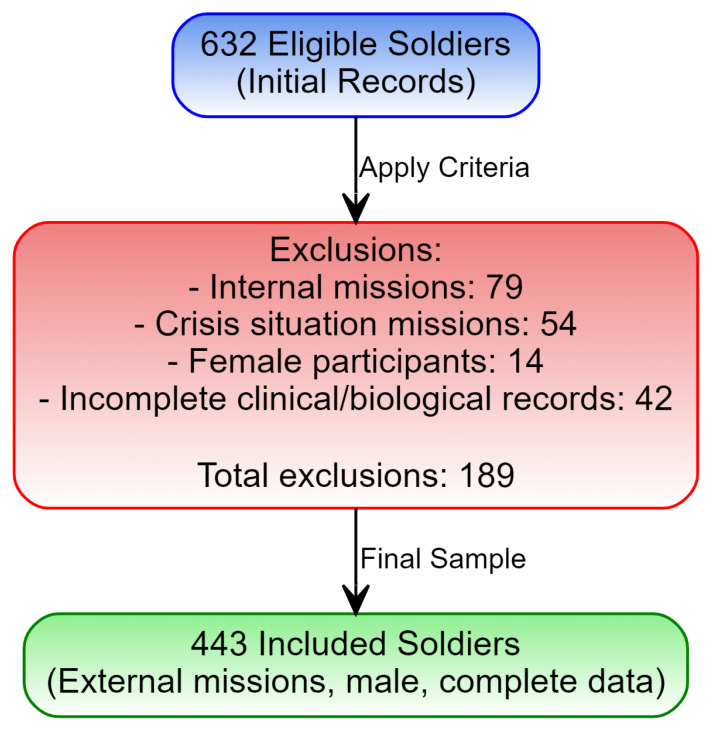
Study population selection flowchart for military personnel.

Initially, 632 eligible military personnel were identified (Figure 1).

After applying stringent inclusion and exclusion criteria, the final sample was refined to 443 participants, ensuring sample homogeneity and the integrity of subsequent analyses.

### 2.2. Inclusion Criteria

The study considered only soldiers who had participated in international missions aimed at defending national territory within the framework of collective defense alliances (e.g., NATO), maintaining international peace and stability, or engaging in operations against international terrorism. To ensure the robustness and reliability of the analyses, only complete clinical and biological records were included—adhering to institutional protocols available in the electronic database of the Military Hospital “Dr. Ștefan Odobleja”.

### 2.3. Exclusion Criteria

Service members who participated exclusively in domestic missions were excluded, as the study was specifically designed to evaluate the impact of a changing operational environment and its associated effects. Furthermore, individuals participating in missions under crisis conditions (such as emergency interventions or periods of international tension) or in active combat operations during wartime were not included, given that both biological and psychoemotional parameters are profoundly influenced in such contexts. Furthermore, the female subgroup was omitted from the analysis due to the very limited number of cases (14 women), which could potentially skew the statistical results. As a result, the final cohort comprised only male military personnel who had participated in overseas missions under comparable conditions and timeframes.

### 2.4. Data Collection

For each participant, clinical, biological, and demographic data were extracted from the electronic database of the Military Hospital “Dr. Ștefan Odobleja” in Craiova, with the objective of capturing changes from the point of deployment to the moment of return. The parameters selected for analysis included inflammatory markers, indicators of oxidative stress, and other biomarkers pertinent to a comprehensive evaluation of general health status. Rigorous validation procedures were implemented that involved independent verification and cross-correlation of data, and any records that were found to be inconsistent or incomplete were excluded from the study. This research protocol received approval from the Medical and Ethics Council of the Military Hospital “Dr. Ștefan Odobleja” in Craiova, in compliance with international regulations for medical research and the Declaration of Helsinki guidelines (approval number A/OR1-972/27 September 2023). Furthermore, ethical clearance was granted by the Ethics Committee of the University of Medicine and Pharmacy in Craiova (approval number 355/17 September 2024).

### 2.5. Statistical Analysis

Data processing was performed using SPSS (version 26.0, IBM Corporation, Armonk, NY, USA), Python (version 3.11) and specialized libraries (pandas, numpy, scikit-learn, imbalanced-learn, Matplotlib (version 3.7.3), and Seaborn (version 0.12.2)). Continuous variables that follow a normal distribution are presented as means ± standard deviations, whereas categorical variables are reported as percentages. Group comparisons were performed using the independent samples *t*-test, the Mann–Whitney U test for non-parametric data, and Fisher’s Exact Test for categorical variables with low frequencies.

**Figure 2:**
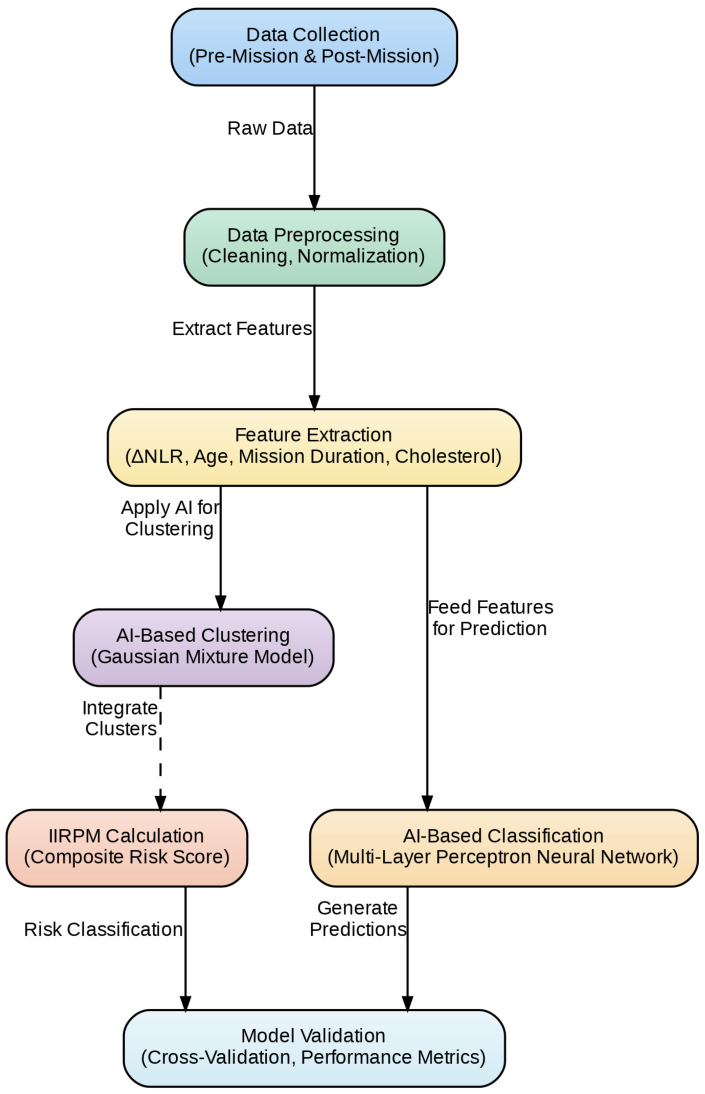
Overview of the research framework for post-mission risk assessment.

The methodological diagram (Figure 2) provides a clear overview of the study’s workflow. Initially, data were collected both pre- and post-mission and then preprocessed through cleaning and normalization. Key features—including ΔNLR, age, mission duration, and cholesterol—were extracted from the dataset.

These features were used in two parallel AI-based analyses: first, a Gaussian mixture model was applied to segment the participants into distinct clusters, forming the basis for computing the Post-Mission Integrated Risk Score (IIRPM); second, the same features were fed into a multi-layer perceptron neural network to classify the profiles, with its performance evaluated via cross-validation. This dual approach—clustering with GMM and classification with MLP—ensures a robust assessment of inflammatory risk in military personnel.

#### 2.5.1. Data Preprocessing

Before advanced analysis, the dataset was meticulously cleaned and standardized. Continuous variables were normalized using the MinMaxScaler method to constrain them within a unit interval. Categorical variables were appropriately encoded using either one-hot or label encoding, depending on their nature. Incomplete records were excluded, and outlier values were rigorously evaluated and removed if they could not be justified on methodological or clinical grounds.

#### 2.5.2. Data Augmentation and Balancing

To address the imbalance among different military profiles (identified later in the clustering analysis) and to prevent the model from being disproportionately influenced by underrepresented classes, an oversampling strategy using SMOTE (Synthetic Minority Over-Sampling Technique) was employed. This algorithm generated new synthetic instances based on existing characteristics, contributing to a more balanced training set and reducing the risk of overfitting to the majority classes.

This data augmentation method was integrated into a processing pipeline that encompassed both the preprocessing steps and the neural network training.

#### 2.5.3. Neural Network Model

**Figure 3:**
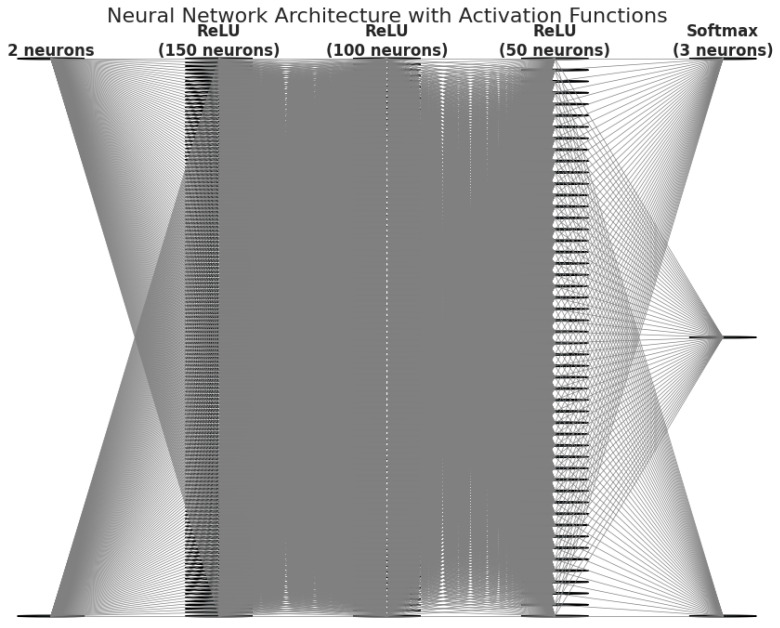
Neural network architecture for post-mission risk classification.

An MLP was developed to classify military personnel according to their inflammatory and oxidative stress profiles. The input variables used to train the MLP model were specifically the normalized values of mission duration and the post-mission neutrophil-to-lymphocyte ratio, as these features demonstrated the strongest discriminatory power in the clustering analysis (Figure 3).

The neural network architecture consisted of an input layer with two neurons corresponding to these features, three hidden layers with 150, 100, and 50 neurons, respectively (all utilizing the ReLU activation function), and an output layer with three neurons corresponding to the identified clusters. A Softmax function was applied to the output to facilitate multiclass classification. Optimization was achieved using the Adam optimizer with an adaptive learning rate, complemented by regularization techniques, namely early stopping and L2 penalization, to ensure both the stability and robustness of the model.

#### 2.5.4. Model Validation

Due to the relatively small size of the dataset (443 military personnel), we opted for 5-fold cross-validation rather than 10-fold CV, as this approach ensures that each fold contains approximately 88 samples, thereby providing more stable and reliable performance estimates compared to having only about 44 samples per fold. Key performance metrics included accuracy, precision, recall, F1-score, the Matthews correlation coefficient, and Cohen’s Kappa. This rigorous validation framework was instrumental in detecting any signs of overfitting and verifying that the model was capturing meaningful associations between the variables related to inflammation and oxidative stress. Our examination of the confusion matrix and learning curves further illuminated the structure of misclassifications and the complexity inherent in the dataset.

#### 2.5.5. Capturing Variable Interactions

The neural network was specifically engineered to uncover complex, nonlinear interactions among clinical and biochemical variables. This design allowed for the detection of synergistic effects, such as the amplification of inflammation under conditions of increased oxidative stress, as well as antagonistic interactions that might mitigate the impact of certain pro-inflammatory factors.

#### 2.5.6. Advanced Clustering for Profile Identification

An initial analysis of all available variables revealed that mission duration and the post-mission neutrophil-to-lymphocyte ratio provided the strongest discrimination between participant profiles. Post-mission NLR was chosen because it directly reflects the intensity of the inflammatory response induced by prolonged operational stress, while mission duration was included as it directly affects the level of exposure to stressors, exhibiting clear differences across subgroups. Together, these two parameters enabled a robust and clinically relevant segmentation of the military personnel.

To delineate clinically homogeneous subgroups, the participants were segregated using a Gaussian mixture model (GMM) clustering algorithm. Both features were normalized using the MinMaxScaler to ensure comparability. The GMM was configured with three components (n_components = 3) to capture the natural heterogeneity within the data, with a random_state set to 42 for reproducibility and n_init equal to 10 to ensure robust initialization. The clustering solution was validated by computing the silhouette score, which confirmed that a three-cluster configuration provided a good separation of profiles. Furthermore, a dimensionality reduction through Principal Component Analysis (PCA) enabled a two-dimensional visualization of the clusters, thereby corroborating the internal consistency of the clustering results.

Furthermore, dimensionality reduction through Principal Component Analysis (PCA) enabled a two-dimensional visualization of the clusters, which corroborated the internal consistency of the clustering results.

#### 2.5.7. Calculation of the Post-Mission Integrated Risk Index (IIRPM)

The Post-Mission Integrated Risk Index (IIRPM) was computed by summing the individual scores of four clinically relevant parameters: ΔNLR, age, mission duration, and post-mission cholesterol level. Each parameter was assigned a score ranging from 0 to 2 points based on clinically meaningful thresholds derived from the statistical analyses (see Section 3, Table 6). The total IIRPM score thus spans from 0 (minimal risk) to 8 (maximum risk), facilitating straightforward risk stratification.

## 3. Results

### 3.1. Descriptive Analysis of Hematological and Biochemical Parameters

Within the final cohort of 443 military personnel, comparative analyses of hematological and biochemical data obtained at deployment and upon return revealed statistically significant variations in several parameters (see Table 1).

Paired *t*-tests were used to assess differences in mean values before and after missions. In particular, the neutrophil count increased significantly (from 3.61 ± 1.11 to 4.29 ± 1.57; *p* < 0.0001), suggesting an augmented immune response associated with operational stress. In parallel, a significant decrease in hematocrit was observed (*p* = 0.0136), potentially indicative of mild hemodilution. Furthermore, triglyceride levels showed a marked elevation (from 121.96 ± 63.27 mg/dL to 150.15 ± 107.00 mg/dL; *p* < 0.0001), which may reflect metabolic influences on the inflammatory response.

In terms of derived inflammatory indices, NLR increased significantly—from 1.62 ± 0.54 to 1.90 ± 0.80 (*p* < 0.0001)—signaling a more pronounced systemic immune activation. Similar trends were observed in other indices, such as the Systemic Inflammation Response Index (SIRI) and the Systemic Immune-Inflammation Index (SII), further supporting the presence of increased oxidative stress and an exaggerated inflammatory response (Table 2).

Marked increases in NLR and SIRI support the hypothesis of an amplified inflammatory response after deployment, while the cumulative inflammatory index (IIC) demonstrated a significant rise (from 1.86 ± 0.72 to 2.14 ± 0.94). However, the evaluation of individual parameters alone proved insufficient to precisely stratify military personnel by degree of impact. Consequently, a clustering approach was subsequently employed to identify distinct inflammatory and metabolic response patterns.

### 3.2. Selection of the Inflammatory Marker and Development of the NLR-Based Risk Score

To elucidate how operational conditions influence the inflammatory response, several hematological indices were analyzed, namely NLR, SIRI, SII, and IIC, with a primary focus on NLR measured before and after deployment. Pearson’s correlation analysis revealed that while SII exhibited a very high correlation (r = 0.7094), its limited variability reduced its sensitivity to detect acute changes. In contrast, NLR showed a moderate correlation (r = 0.6299) with a highly significant difference between the pre- and post-deployment values (*p* = 1.43 × 10^−50^) (Table 3).

**Figure 4:**
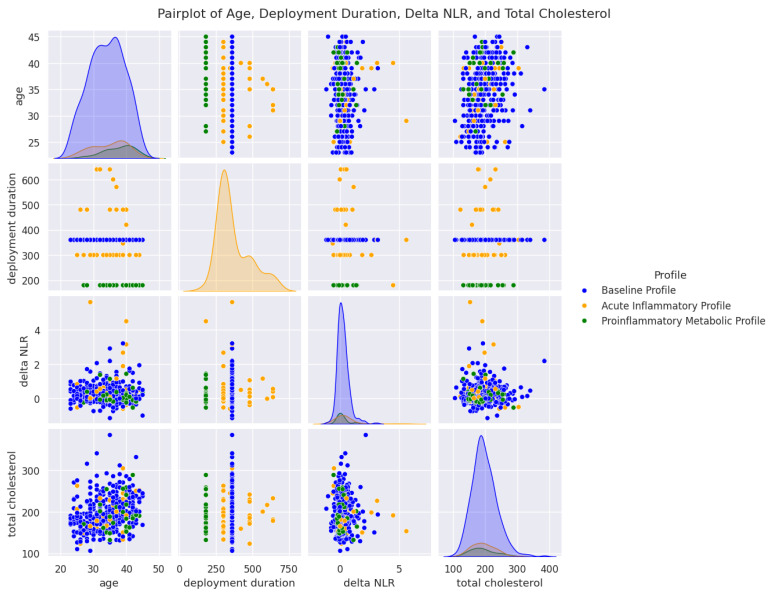
Distribution and intercorrelations among ΔNLR, age, mission duration, and post-mission cholesterol.

Based on these findings, a new variable—ΔNLR—was defined as the difference between post-deployment and pre-deployment NLR values (NLR_3_—NLR_2_). This derived parameter serves as an individualized indicator of change, allowing for a more nuanced classification of participants according to their response to operational stress. To further explore the interplay among ΔNLR, age, mission duration, and post-mission cholesterol levels, a pair plot was generated (Figure 4), which confirmed the presence of potentially complex interrelationships between these variables.

Subsequently, a regression model was constructed with post-deployment NLR (NLR__pred_) as the dependent variable and ΔNLR, age, mission duration, and cholesterol measured upon return as predictors. The model formulation is as follows:

NLR_pred_ = β_0_ + β_1_ × ΔNLR + β_2_ × Age + β_3_ × Mission Duration + β_4_ × Post-Mission Cholesterol

The coefficients obtained (Table 4) indicated that each predictor contributed significantly to the variations observed in the NLR after deployment. Notably, ΔNLR was particularly effective in capturing acute inflammatory changes, while post-deployment cholesterol emerged as a significant predictor due to its correlation with elevated inflammatory risk.

The post-deployment data were then subjected to clustering analysis, which segregated the participants into three distinct groups: Cluster 0 (“Basal Profile”), Cluster 1 (“Acute Inflammatory Profile”), Cluster 2 (“Metabolic Proinflammatory Profile”).

In Cluster 1, the post-deployment NLR reached its highest value (2.53 ± 1.31), with ΔNLR increasing by +0.70 and associated with elevated cholesterol levels (210.30 ± 40.8 mg/dL). In contrast, Cluster 2 exhibited a more moderate increase in NLR (+0.31), but this was accompanied by significantly higher cholesterol levels and a higher mean age (Table 5).

These results indicate that personnel in Cluster 1 show a more robust inflammatory response, while those in Cluster 2 tend to display a metabolic profile that predisposes them to inflammation.

**Figure 5:**
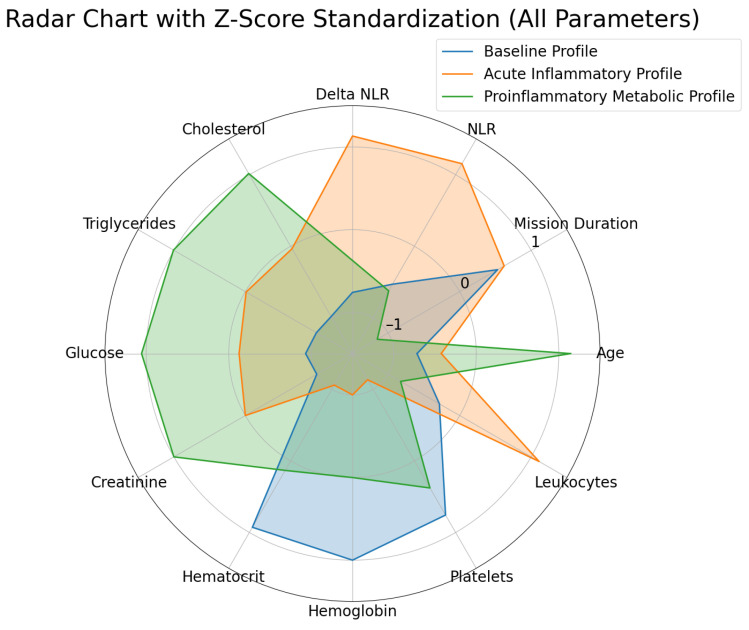
Radar chart of profiles with parameter standardization using Z-score.

To ensure comparability between different measurement units, all parameters were standardized using the Z-Score method. This standardization, illustrated in the radar chart (Figure 5), allowed for a fair comparison by placing all variables on a common scale, thus facilitating a clear visualization of the differences and variations among the identified profiles.

Based on these conclusions, the Post-Mission Integrated Risk Index (IIRPM) was developed, with a composite score calculated by summing the individual scores of four clinically relevant parameters: ΔNLR, age, mission duration, and post-mission cholesterol level. Each parameter was assigned a score ranging from 0 to 2 points according to clearly defined intervals reflecting clinical relevance (Table 6).

Specifically, higher ΔNLR values indicate acute inflammatory responses, increased age and elevated cholesterol levels reflect greater cardiometabolic risk, and longer mission duration represents extended exposure to operational stressors. The sum of these individual scores yields a total IIRPM score ranging from 0 (minimal risk) to 8 (maximum risk), facilitating clear risk stratification (Table 7).

Elevated cholesterol levels were incorporated into the index based on their significantly different distribution between clusters and their association with NLR changes, suggesting a metabolic environment conducive to inflammation.

Through this integrative approach, a practical tool has been developed that can be readily implemented in military clinical practice. This instrument facilitates the rapid identification of personnel at high risk for acute inflammation and metabolic imbalances post-deployment, thereby supporting personalized preventive interventions and continuous health monitoring in the context of military operations.

### 3.3. Evaluation of Model Performance

The MLP neural network, designed to classify military personnel according to their post-mission inflammatory and metabolic profiles, was rigorously evaluated using several key performance metrics: accuracy, precision, recall, F1-score, the Matthews correlation coefficient (MCC), and Cohen’s Kappa. Together, these metrics provide a robust assessment of the model’s ability to detect physiological and biochemical alterations associated with operational stress.

### 3.4. Performance on the Test Set

**Figure 6:**
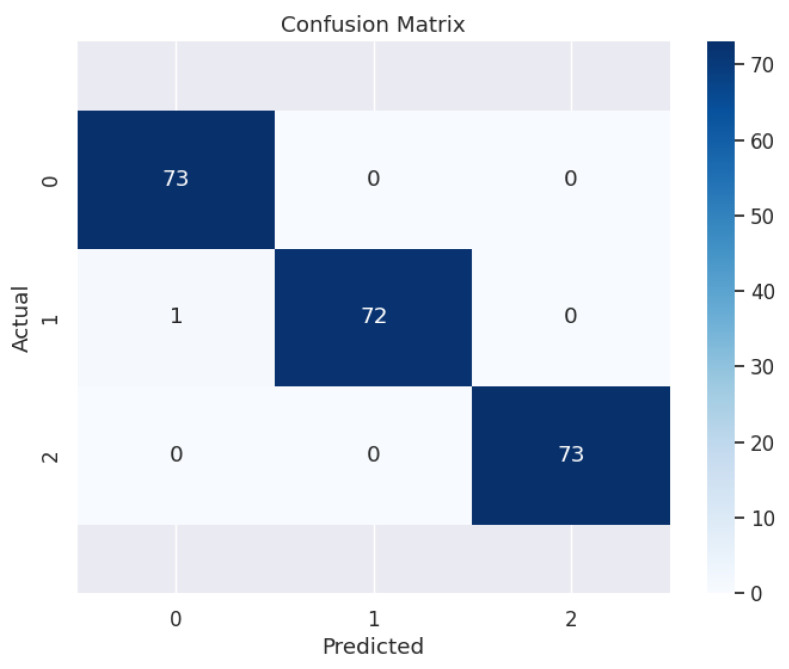
Confusion matrix of the neural network model.

When applied to the test dataset, the model achieved a perfect accuracy of 100%. The confusion matrix (Figure 6) clearly demonstrates that almost all instances were correctly classified, with only one misclassification, where a case belonging to Profile 1 was incorrectly identified as Profile 0.

Both precision and recall were 100%, and the F1 score was recorded at 1.00, highlighting the exceptional performance of the model. Furthermore, the MCC was calculated at 0.99, indicating an extremely strong correlation between the predictions of the model and the actual labels, while Cohen’s Kappa also reached 0.99, reflecting an excellent level of agreement.

### 3.5. Learning Curve and Training Stability

**Figure 7:**
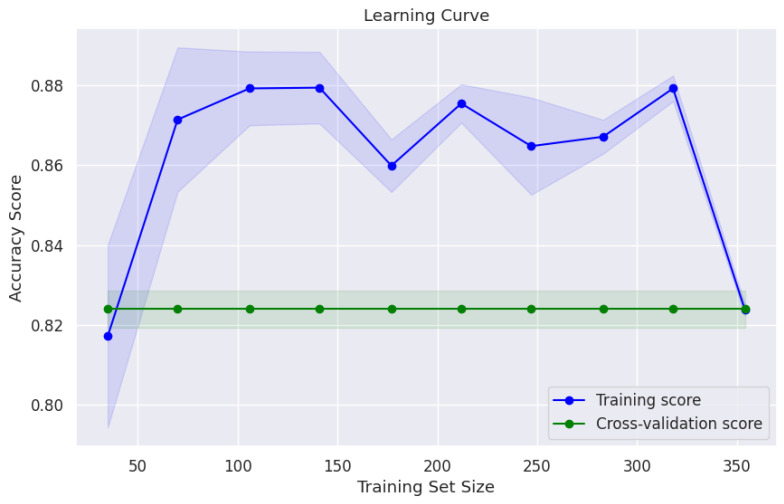
Learning curve of the neural network.

To assess the stability of the training and the overall efficiency of the learning process, the neural network learning curve was analyzed (Figure 7).

This curve provides insight into the progression of the model’s performance over time, illustrating its ability to generalize to unseen data. The observed reduction in the training accuracy at the largest training set size (final data point) is due to averaging the performance across cross-validation folds, reflecting variations inherent to smaller training subsets within certain folds. At smaller subset sizes, the training performance metric may appear elevated due to easier memorization of fewer instances; however, as the dataset size approaches its maximum, the average training accuracy reflects the consistent generalization across all folds, stabilizing toward the true generalization performance. Thus, this observed fluctuation does not indicate decreased model learning capacity but rather highlights the model’s behavior when averaging performance across varied subsets within the cross-validation procedure. In particular, the accuracy of the training experienced a rapid increase before stabilizing at approximately 88%. In parallel, the accuracy of the validation remained steady at around 82%, suggesting an optimal balance that minimizes the risks of both under- and over-fitting. The relatively narrow confidence band around the learning curve further supports the robustness and consistency of the model.

### 3.6. Model Validation via Cross-Validation

A 5-fold cross-validation approach was employed to further verify the model’s generalizability. Across the folds, the accuracy of the model ranged from 93.26% to 100%, with an average accuracy of approximately 97% and a standard deviation of 2%. The weighted precision and recall were recorded at 98% and 97%, respectively, which confirms the robustness of the classification performance.

**Figure 8:**
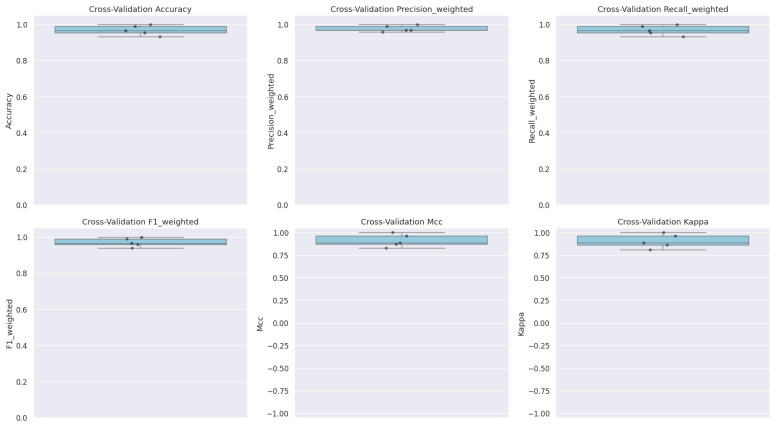
Cross-validation scores for accuracy, precision, recall, and F1-score.

The MCC values in the folds varied between 0.828 and 1.000, yielding an average of 0.91 (standard deviation = 0.06). Similarly, Cohen’s Kappa values ranged from 0.811 to 1.000, with an average of 0.91 (standard deviation = 0.07). These high values strongly indicate a robust correlation between predicted classifications and true labels and underscore a high level of agreement between the predictions of the model and the observed class distribution between the military personnel (Figure 8).

**Figure 9:**
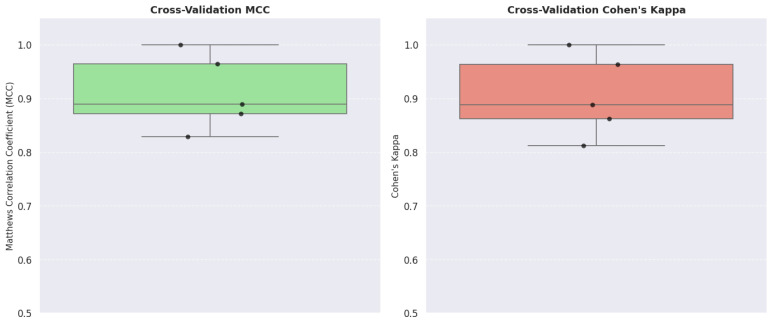
MCC and Cohen’s Kappa across the 5 folds.

More specifically, the MCC values for each fold were 0.964, 0.828, 1.000, 0.871, and 0.888, while the corresponding Cohen’s Kappa values were 0.963, 0.811, 1.000, 0.862, and 0.888. These figures further confirm the strong predictive performance of the model and its ability to reliably capture the distribution of the inflammatory and metabolic changes observed after deployment (Figure 9).

## 4. Discussion

This study was designed to advance the evaluation of oxidative stress and inflammation in military personnel returning from international missions by employing an integrative approach that combines rigorous statistical analysis with advanced artificial intelligence techniques. Based on data collected from 443 military subjects over a ten-year period, the findings offer a detailed account of how prolonged exposure to hostile operational conditions affects hematological and metabolic parameters. Furthermore, by segmenting the data into three distinct clusters and constructing a predictive model, the Post-Mission Integrated Risk Index (IIRPM) was derived, which integrates NLR and its change (ΔNLR) with key clinical factors such as age, post-mission cholesterol levels, and duration of the mission.

### 4.1. Cluster Segmentation and Biological Significance

Cluster analysis revealed three distinct response profiles that underscore the individual variability between military personnel. The first cluster, labeled the “Reference Profile” and comprising approximately 80% of the sample, is characterized by relative immunologic stability, with only a minimal increase in ΔNLR (+0.21 ± 0.44). This observation is consistent with previous literature suggesting that, in the absence of acute inflammatory stress, the NLR remains close to baseline values [17]. In addition, earlier studies on military populations have shown that most individuals return to normal physiological parameters after a mission, without major inflammatory alterations [18].

In contrast, the “Acute Inflammatory Response” cluster (approximately 13.1% of participants) is marked by a significant increase in ΔNLR (+0.70 ± 1.07), suggesting an intense inflammatory reaction and potentially increased oxidative stress. This response is frequently associated with sustained physical exertion and intense psychological stress—factors known to exacerbate systemic inflammation and increase the risk of cardiovascular events [19,20].

The third cluster, termed the “Metabolic Proinflammatory Profile” (7% of the sample), is characterized by a moderate ΔNLR increase (+0.31 ± 0.90) combined with elevated cholesterol levels and a higher mean age. These findings imply an underlying predisposition to chronic inflammation and cardiometabolic risk, supporting the hypothesis that aging and dyslipidemia may synergistically sustain a proinflammatory state [21,22,23].

### 4.2. Relevance of ΔNLR and Its Correlation with the Cumulative Inflammatory Index

The differential analysis of NLR, captured by ΔNLR, emerged as a central parameter in quantifying the inflammatory response to operational stress. The significant increase in ΔNLR, particularly observed in the “Acute Inflammatory Response” cluster, highlights the marker’s sensitivity to rapid changes induced by intense physical and psychological stress [24]. Furthermore, the positive correlation between ΔNLR and the Cumulative Inflammatory Index (IIC) indicates that dynamic monitoring of this marker may effectively predict the long-term impact of operational stress on an individual’s inflammatory status [3,25]. Mechanistically, neutrophils activation results in increased production of reactive oxygen species (ROS), thereby intensifying oxidative stress, while the concomitant reduction in lymphocyte count contributes to immune imbalance [26,27,28,29].

### 4.3. Predictive Model and the Role of Artificial Intelligence

Beyond descriptive and cluster analyses, a predictive model was developed using an MLP neural network to classify inflammatory profiles based on ΔNLR, age, mission duration, and post-mission cholesterol levels. The model achieved 100% accuracy on the test set and an average of 97% accuracy in cross-validation, underscoring the robustness of AI in capturing complex, non-linear relationships among clinical and biological variables [5,30,31]. This machine learning approach offers a distinct advantage over traditional statistical methods by simultaneously processing large volumes of data and detecting subtle patterns that might otherwise be overlooked. When implemented in a digital application, this model can enable medical personnel to quickly compute the IIRPM score using standard clinical data, thus facilitating prompt and informed clinical decision-making.

### 4.4. Age, Mission Duration, and Cholesterol: Foundations of the IIRPM

The linear regression analysis confirmed the cumulative impact of ΔNLR, age, mission duration, and post-mission cholesterol on the inflammatory response. With advancing age, the process of “inflammaging”—characterized by a decline in antioxidant capacity and increased low-grade inflammation—can exacerbate the inflammatory response, particularly when compounded by dyslipidemia [32]. Elevated cholesterol levels, especially when associated with reduced HDL levels, further intensify oxidative stress and may lead to endothelial dysfunction, promoting atherosclerosis [33,34]. In addition, extended mission durations reflect prolonged exposure to adverse environmental conditions such as high temperatures, pollution, or sleep deprivation, all of which deplete antioxidant reserves and raise inflammatory markers [1,35,36]. Incorporating these variables into the IIRPM score provides a comprehensive assessment of post-mission inflammatory risk.

### 4.5. Clinical Implications and Operational Context

The IIRPM score represents a novel, practical tool for military medicine, enabling the rapid stratification of inflammatory and oxidative risks. In operational contexts where rapid response and resource allocation are critical, this score can identify individuals who require focused monitoring and personalized intervention. Personnel with elevated IIRPM scores can benefit from intensive recovery protocols, targeted nutritional interventions (e.g., cholesterol reduction and antioxidant supplementation), and psychological support to mitigate long-term cardiovascular and metabolic complications [21,22,37,38,39,40,41]. The integration of this AI-based evaluation into digital platforms offers the potential for real-time clinical decision-making, especially in resource-limited operational settings.

### 4.6. Influence of Environmental and Psychological Factors

In addition to biological and metabolic parameters, environmental and psychological factors play a crucial role in shaping the post-mission inflammatory response. Exposure to extreme conditions, such as pollution, radiation, or even microgravity during space missions, can catalyze oxidative stress [42,43,44]. Simultaneously, chronic psychological stress, anxiety, and existential fears can activate the hypothalamic–pituitary–adrenal axis, leading to increased glucocorticoid secretion that further amplifies inflammation and oxidative stress [29,40]. These findings underscore the importance of a multidisciplinary approach in assessing military health, integrating environmental and psychological factors into comprehensive preventive and intervention strategies.

### 4.7. Study Limitations

Despite its promising findings, this study has several limitations. First, the sample consisted exclusively of male military personnel, which may limit the generalizability of the results to the entire military population, including female service members. Second, the analysis focused on international missions conducted under relatively homogeneous operational conditions, excluding crisis situations or domestic deployments where stressors might be more variable or severe. Furthermore, the study did not include detailed psychoemotional assessments, such as measures of anxiety or post-traumatic stress, nor did it incorporate genetic or epigenetic markers that might influence the inflammatory response to operational stress. These limitations suggest important avenues for future research aimed at further refining and expanding the IIRPM model.

### 4.8. Implications and Future Perspectives

This study underscores the critical importance of an integrated evaluation of hematological, metabolic, and clinical parameters in quantifying post-mission inflammatory risk. Through cluster segmentation, differential analysis of ΔNLR, and the integration of variables such as age, mission duration, and cholesterol levels, an innovative risk score, the IIRPM, was developed, which can guide personalized preventive interventions in military medicine. The application of advanced AI techniques not only optimizes data analysis and minimizes interpretative errors but also opens up new possibilities for expanding this research. Future studies should consider incorporating additional variables, such as psychological and genetic factors, and validate the model in diverse populations. Furthermore, the development of digital applications based on this model could enable the rapid integration of the IIRPM score into military health monitoring systems, facilitating swift and effective management of inflammatory and metabolic risks.

## 5. Conclusions

The findings of this study underscore the complexity of the inflammatory and oxidative responses in military personnel returning from international missions, revealing the presence of at least three distinct profiles: a baseline profile marked by immunological stability, an acute inflammatory response profile, and a metabolic pro-inflammatory profile. In this context, ΔNLR emerged as an exceptionally sensitive marker, capable of rapidly detecting both immune alterations induced by operational stress and potential cardiometabolic risks.

The innovative approach, which integrates multiple parameters—including age, mission duration, and cholesterol levels—into a predictive model based on advanced artificial intelligence techniques, demonstrated outstanding classification performance. The robust results obtained through modern statistical segmentation and validation with an MLP neural network support the reliability and clinical utility of the IIRPM score as an effective tool for triaging and monitoring post-mission risk.

From a practical point of view, the IIRPM score can be implemented as a digital application within military health systems, thus facilitating the early identification of vulnerable personnel and guiding personalized preventive interventions aimed at reducing oxidative stress and managing inflammation. Consequently, the results of this study not only contribute significantly to our understanding of complex biological mechanisms but also open new avenues for research and clinical applications.

In general, effective health management among military personnel requires an integrated approach that combines careful monitoring of inflammatory biomarkers with comprehensive assessments of demographic, metabolic, psychological, and environmental factors. Further validation of the IIRPM score through multicenter, expansive, and longitudinal studies will consolidate the relevance of this innovative instrument and establish a new standard in military medicine, tailored to the multifaceted and often unpredictable challenges encountered in operational theaters.

## Figures and Tables

**Table 1:** Hematological and biochemical parameters before and after deployment.

Biological Parameter	Mean ± SD (Pre-Mission)	Mean ± SD (Post-Mission)	*p*-Value (*t*-Test)
Basophils (×10^3^/µL)	0.03 ± 0.02	0.03 ± 0.02	0.4311
Total Cholesterol (mg/dL)	194.69 ± 37.16	196.69 ± 38.25	0.4275
Creatinine (mg/dL)	0.92 ± 0.18	0.91 ± 0.15	0.8151
Eosinophils (×10^3^/µL)	0.29 ± 2.18	0.19 ± 0.12	0.3613
Glucose (mg/dL)	96.51 ± 8.62	96.69 ± 8.12	0.7446
Neutrophils (×10^3^/µL)	3.61 ± 1.11	4.29 ± 1.57	<0.0001 *
Hematocrit (%)	44.74 ± 3.94	44.20 ± 2.46	0.0136 *
Hemoglobin (g/dL)	15.33 ± 1.70	15.43 ± 0.95	0.2705
Lymphocytes (×10^3^/µL)	2.51 ± 4.03	2.37 ± 0.62	0.4668
MCH (pg)	30.00 ± 2.49	30.19 ± 1.51	0.1824
MCHC (g/dL)	34.24 ± 2.76	34.92 ± 0.91	<0.0001 *
MCV (fL)	88.09 ± 8.24	86.47 ± 4.01	0.0002 *
Monocytes (×10^3^/µL)	1.30 ± 14.72	0.66 ± 0.19	0.3637
MPV (fL)	10.82 ± 1.21	10.67 ± 0.92	0.0433 *
PCT (%)	0.28 ± 0.61	0.24 ± 0.05	0.2709
Platelets (×10^3^/µL)	232.72 ± 55.13	231.59 ± 50.56	0.7495
RBC (×10⁶/µL)	5.20 ± 2.10	5.12 ± 0.37	0.4241
RDW (%)	13.14 ± 3.59	13.05 ± 0.84	0.6034
AST (U/L)	24.69 ± 7.75	23.05 ± 8.63	0.0029 *
ALT (U/L)	29.28 ± 13.45	27.43 ± 13.65	0.0416 *
Triglycerides (mg/dL)	121.96 ± 63.27	150.15 ± 107.00	<0.0001 *
ESR (mm/hr)	8.02 ± 5.12	7.33 ± 5.01	0.0444 *
WBC (×10^3^/µL)	6.74 ± 1.57	7.54 ± 1.96	<0.0001 *

* statistically significant (*p* < 0.05).

**Table 2:** Inflammatory markers before and after deployment.

Inflammatory Marker	Mean ± SD (Pre-Mission)	Mean ± SD (Post-Mission)	*p*-Value (*t*-Test)
NLR	1.62 ± 0.54	1.90 ± 0.80	<0.0001 *
PLR	105.14 ± 30.98	103.46 ± 32.80	0.4336
MLR	0.27 ± 0.18	0.29 ± 0.09	0.0853
SIRI	0.99 ± 0.54	1.28 ± 0.72	<0.0001 *
SII	377.79 ± 163.92	443.79 ± 234.42	<0.0001 *
IIC	1.86 ± 0.72	2.14 ± 0.94	<0.0001 *

* statistically significant (*p* < 0.05).

**Table 3:** Pearson correlation analysis for inflammatory markers.

Marker	Pearson Correlation (r)	*p*-Value
NLR_2_—NLR_3_	0.6299	*p* < 0.0001 (11.43 × 10^−50^)
SIRI_2_—SIRI_3_	0.4938	*p* < 0.0001 (9.87 × 10^−29^)
SII_2_—SII_3_	0.7094	*p* < 0.0001 (2.67 × 10^−69^)
IIC_2_—IIC_3_	0.5747	*p* < 0.0001 (1.79 × 10^−40^)

_2_—pre-deployment; _3_—post-deployment.

**Table 4:** Regression coefficients for the NLR-based model.

Variable	Coefficient (β)	Standard Error	*p*-Value
Intercept	−1.20	0.33	<0.001 *
ΔNLR	2.05	0.42	<0.001 *
Age (years)	0.07	0.03	0.005 *
Mission Duration (days)	0.0048	0.0018	0.017 *
Post-Mission Cholesterol (mg/dL)	0.011	0.003	0.004 *

* statistically significant (*p* < 0.05).

**Table 5:** Comparison of key biological parameters across clusters.

Parameter	Cluster 0 (*n* = 354)	Cluster 1 (*n* = 58)	Cluster 2 (*n* = 31)	*p*-Value
Age (years)	34.00 ± 5.4	34.60 ± 5.3	37.80 ± 4.5	0.0003
Mission Duration (days)	360.00 ± 0.7	369.80 ± 98.6	180.00 (fix)	<0.0001
NLR (post-deployment)	1.80 ± 0.57	2.53 ± 1.31	1.76 ± 1.12	<0.0001
ΔNLR	0.21 ± 0.44	0.70 ± 1.07	0.31 ± 0.90	<0.0001
Cholesterol (mg/dL)	196.50 ± 39.3	210.30 ± 40.8	225.60 ± 46.6	<0.0001
Triglycerides (mg/dL)	140.30 ± 86.5	158.20 ± 68.1	176.70 ± 78.5	<0.0001
Glucose (mg/dL)	96.80 ± 8.12	98.30 ± 8.62	100.50 ± 9.0	<0.0001
Creatinine (mg/dL)	0.91 ± 0.14	0.93 ± 0.11	0.95 ± 0.15	0.0080
Hematocrit (%)	44.20 ± 2.46	43.70 ± 2.50	44.00 ± 2.45	0.0020
Hemoglobin (g/dL)	15.40 ± 0.95	15.20 ± 1.00	15.30 ± 0.98	0.1250
Platelets (10^3^/µL)	231.60 ± 50.56	228.10 ± 48.98	230.90 ± 51.20	0.0007
Leukocytes (10^3^/µL)	7.54 ± 1.96	8.62 ± 2.00	7.12 ± 1.75	<0.0001

**Table 6:** Calculation of the Post-Mission Integrative Inflammatory Risk Index (IIRPM).

Parameter	Interval/Values	Assigned Score
ΔNLR (Delta NLR)	<0.2	0
	0.2–0.5	1
	≥0.5	2
Age (years)	<35	0
	35–40	1
	>40	2
Mission Duration (days)	<300	0
	300–360	1
	≥360	2
Post-Mission Cholesterol (mg/dL)	<190	0
	190–220	1
	>220	2

**Table 7:** Classification of inflammatory risk based on IIRPM Score.

Total Score	Risk Level	Interpretation
<2 points	Low	Minimal risk; no significant concerns.
3–4 points	Moderate	Present risk; warrants monitoring.
≥5 points	High	Elevated risk; requires enhanced monitoring and potential clinical intervention.

## Data Availability

The authors declare that the data of this research are available from the corresponding authors, upon reasonable request.
